# Supplementary material for: Conditional chemoconnectomics (cCCTomics) as a strategy for efficient and conditional targeting of chemical transmission
Source: eLife. 2024 Apr 30;12:RP91927. doi: 10.7554/eLife.91927 (PMC11060718; doi:10.7554/eLife.91927)
Supplement: Figure 3—source data 3. [file elife-91927-fig3-data3.zip › Figure 3 source data3 part 2.zip!/Figure 3-Source data 3-part2/1052-13-CRISPResso_Report_ejedac2.zip!/CRISPResso_on_1052_13/1c.Alignment_barplot.pdf]

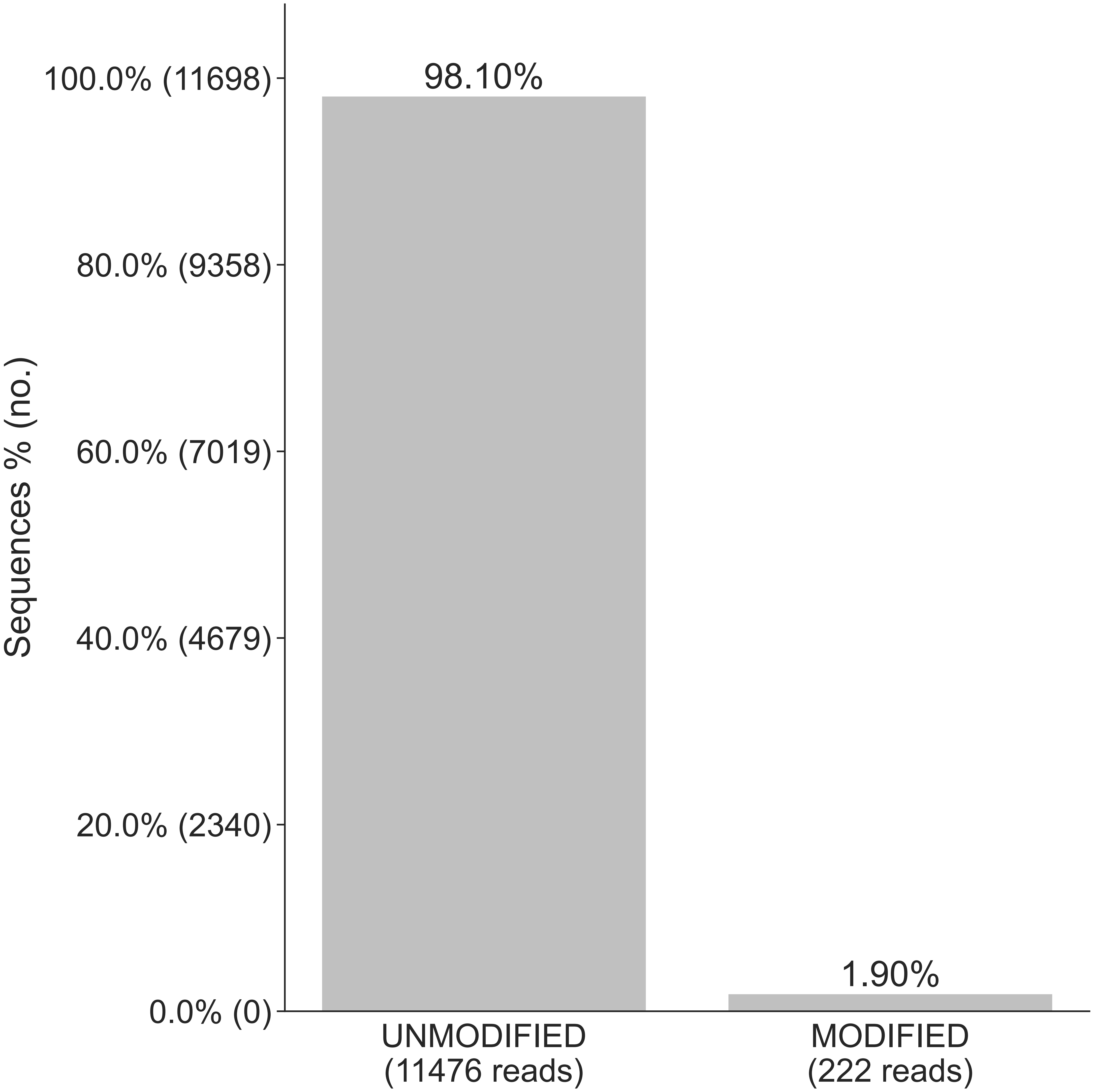

98.10%
1.90%
100.0% (11698)
80.0% (9358)
60.0% (7019)
40.0% (4679)
20.0% (2340)
0.0% (0)
Sequences % (no.)
UNMODIFIED
(11476 reads)
MODIFIED
(222 reads)
